# An Analysis of Global Research Trends in ICU‐Acquired Weakness

**DOI:** 10.1155/emmi/7910286

**Published:** 2026-01-07

**Authors:** Wei Li, Jiadong Wang, Xi Feng

**Affiliations:** ^1^ Department of Emergency, The Fourth Hospital of Changsha (Integrated Traditional Chinese and Western Medicine Hospital of Changsha, Changsha Hospital of Hunan Normal University), Changsha, Hunan, China

**Keywords:** bibliometric analysis, CiteSpace, ICU-acquired weakness, VOSviewer

## Abstract

**Background:**

ICU‐acquired weakness (ICU‐AW) is a severe complication among critically ill patients and represents a common secondary neuromuscular dysfunction syndrome in the intensive care unit (ICU). It significantly impairs patients’ health, functional recovery, and long‐term quality of life.

**Methods:**

We conducted a comprehensive search of the Web of Science Core Collection database for publications related to ICU‐AW, retrieving a total of 1866 records as of September 4, 2025. Using the bibliometric software CiteSpace and VOSviewer, we performed qualitative and quantitative analyses of publication trends, contributing countries, institutions, authors, and keyword co‐occurrence patterns.

**Results:**

The annual number of publications on ICU‐AW has shown a steady upward trajectory, peaking in 2021 with 186 articles. The United States was the most productive country, contributing 512 publications (27.44%). The University of Toronto emerged as the leading institution with 43 publications. The most prolific author was Lars Larsson (Sweden, 34 publications), and the most frequently occurring keyword was “intensive care unit.”

**Conclusion:**

This study is the first to provide a comprehensive bibliometric overview of global research on ICU‐AW. Our findings illuminate current research landscapes, highlight key contributors and thematic foci, and offer novel insights to guide future investigations into the pathophysiology, diagnosis, treatment, and nursing interventions for ICU‐AW. Furthermore, the analysis enables evidence‐based forecasting of emerging research frontiers and evolving trends in this critical field.

## 1. Introduction

ICU‐acquired weakness (ICU‐AW) is a common secondary neuromuscular dysfunction syndrome affecting critically ill patients in intensive care unit (ICU). It can involve both limb and respiratory muscles, with reported prevalence varying widely depending on the study population. Although the underlying pathophysiology remains incompletely understood, it is thought to involve complex structural and functional alterations in both muscle fibers and peripheral neurons. Diagnosis relies on a combination of clinical assessment and electrophysiological tools, each of which carries distinct advantages and limitations. Established risk factors include advanced age, body weight, pre‐existing comorbidities, severity of illness, organ failure, exposure to neuro‐ or myotoxic medications, prolonged immobility, and other ICU‐related interventions [1]. ICU‐AW is associated with significant adverse outcomes, including increased mortality [2], prolonged duration of mechanical ventilation [3], extended ICU and hospital stays, persistent physical dysfunction, and long‐term impairments in health‐related quality of life and prognosis after discharge [4, 5]. To date, the precise pathogenesis of ICU‐AW has not been fully elucidated [6], and no universally accepted diagnostic criteria exist [7]. This article presents a comprehensive bibliometric analysis of the existing literature on ICU‐AW to identify emerging research trends and highlight recent scientific advances in the field.

The term bibliometrics was first introduced by the British scholar Alan Pritchard in 1969 [8]. It refers to the quantitative study of literature systems and their bibliometric characteristics, employing mathematical and statistical methods to analyze patterns and trends in scholarly output [9]. Three foundational laws underpin bibliometric analysis: Bradford’s Law, which describes the scattering of scientific literature across journals; Lotka’s Law, which characterizes the inverse relationship between the number of authors and their publication productivity; and Zipf’s Law, which models the frequency distribution of word usage in scientific texts. Today, bibliometrics has been widely applied across diverse disciplines and plays a pivotal role in identifying research hotspots, mapping intellectual structures, and forecasting emerging trends within a given field.

This study aims to map the current global research landscape and identify key hotspots in ICU‐AW through bibliometric analysis. By employing visualization‐based approaches, this work offers a novel synthesis of the evolving knowledge structure in the field. The primary innovation of this study lies in its use of scientometric visualization to illustrate the trajectory of ICU‐AW research, thereby providing researchers with fresh insights into its pathogenesis, diagnostic criteria, and preventive nursing strategies. Furthermore, the analysis enables evidence‐informed forecasting of future research trends and emerging directions.

## 2. Materials and Methods

### 2.1. Data Source

To identify all publications related to ICU‐AW, we defined a comprehensive search strategy and applied it to the Web of Science Core Collection (WoS CC) database. Bibliometric tools were employed to systematically organize and conduct a thorough analysis of the retrieved literature. Although CiteSpace and VOSviewer are powerful visualization platforms, they do not support the integrated analysis of data from multiple databases. After careful evaluation and comparison of data quality and completeness across sources, we selected the WoS CC as the sole database for retrieval to ensure analytical consistency and reliability.

### 2.2. Eligibility Criteria

We first established explicit inclusion and exclusion criteria to guide the selection of studies. The inclusion criteria were as follows: (1) records retrieved from WoS CC database; (2) content directly relevant to ICU‐AW; (3) publication date up to and including September 4, 2025; (4) document type classified as either “article” or “review”; and (5) publication language limited to English. Conversely, records were excluded if they: (1) originated from databases other than WoS CC; (2) lacked relevance to ICU‐AW; (3) were of document types other than “article” or “review”; or (4) were published in languages other than English.

### 2.3. Search Strategy

In order to retrieve all relevant data as far as possible, by constantly adjusting the search terms and search strategies, we finally locked the search formula as follows: ((((((((((((((TS = (ICU acquired weakness)) OR TS = (Intensive Care Unit acquired weakness)) OR TS = (ICU‐aw)) OR TS = (ICU acquired myasthenia)) OR TS = (Intensive Care Unit acquired myasthenia)) OR TS = (ICU acquired asthenia)) OR TS = (Intensive Care Unit acquired asthenia)) OR TS = (ICU muscle atrophy)) OR TS = (ICU neuromuscular dysfunction)) OR TS = (ICU‐acquired weakness)) OR TS = (critical illness myopathy)) OR TS = (critical illness polyneuropathy)) OR TS = (CIP/CIM)) OR TS = (ICU AND CIP)) OR TS = (ICU AND CIM) OR TS = (CIM AND CIP). After obtaining the preliminary data, we preliminarily screened the literature by setting the literature type (articles and reviews) and language (English).

### 2.4. Data Analysis

We export the retrieved literature into text files in the format of full record and reference and rename the text files as download‐1, download‐2, download‐3, and download‐4. These data were first processed in CiteSpace to remove duplicates, and the deduplicated data were subsequently saved into a newly created folder named “date.” Finally, we obtained 1866 articles. We import the data in the date folder into CiteSpace and VOSviewer again for network visualization analysis and hot word detection of countries, institutions, authors, keywords, and so on.

## 3. Result Analysis

### 3.1. Publication Time Trends

**Figure 1:**
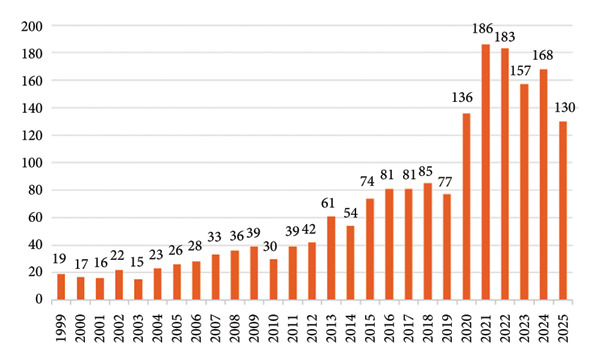
Annual publication outputs from 1999 to 2025.

Figure 1 illustrates the temporal distribution of the 1866 included publications. The earliest article related to ICU‐AW was published in 1999; however, research output remained limited during the initial period, with fewer than 50 publications per year between 1999 and 2012. A gradual increase in publication volume was observed after 2012, and the annual count surpassed 100 for the first time in 2020. The number of publications rose sharply in 2021, peaking at 186—the highest annual output in the study period. Since 2020, the number of publications has remained consistently above 130 per year. Overall, the literature on ICU‐AW demonstrates a sustained upward trend, with annual output stabilizing around 150 publications in recent years. This pattern reflects the growing recognition among researchers of ICU‐AW as a critical factor affecting the health and outcomes of critically ill patients in the intensive care unit.

### 3.2. Country

The 1866 publications originated from 83 distinct countries. Table 1 presents the top 10 countries by publication output, along with their respective betweenness centrality and percentage of total publications. The United States ranked first in publication volume (*n* = 512, 27.44%), followed by Germany (*n* = 191, 10.24%) and China (*n* = 174, 9.33%). Notably, the United States also exhibited the highest betweenness centrality, indicating its pivotal role and well‐established research leadership in the field of ICU‐AW. This suggests that U.S.‐based research not only dominates in quantity but also serves as a central hub in the global collaborative and intellectual network surrounding ICU‐AW.

**Table 1:** The top 10 countries by documents.

Rank	Country	Numbers	Centrality	Ratio (%)
1	UNITED STATES	512	0.46	27.438
2	GERMANY	191	0.12	10.236
3	CHINA	174	0.09	9.325
4	ENGLAND	145	0.12	7.771
5	ITALY	131	0.18	7.02
6	CANADA	129	0.08	6.913
7	FRANCE	126	0.02	6.752
8	AUSTRALIA	108	0.08	5.788
9	JAPAN	95	0.01	5.091
10	NETHERLANDS	95	0.03	5.091

**Figure 2:**
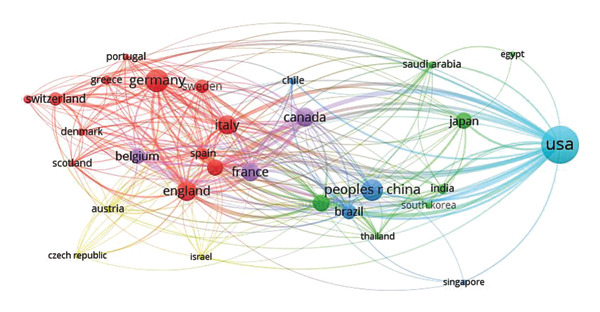
Cooperation network among all countries.

To better visualize and analyze international collaboration patterns, we imported the country‐level data into VOSviewer and applied a minimum threshold of 10 publications per country. This yielded a collaboration network comprising 32 countries that met the inclusion criterion. These 32 countries formed six distinct clusters, illustrated in Figure 2 and differentiated by six colors: red, yellow, blue, green, purple, and another distinct hue. In the network, node size reflects the number of publications from each country, while the thickness of connecting lines indicates the strength of collaborative ties. The largest cluster (red) is centered around England and includes 12 countries, notably Germany, Italy, and the Netherlands. The second‐largest cluster (green) is led by Japan and encompasses seven countries, including Australia, South Korea, and India. A third prominent cluster (blue) is anchored by China, with strong collaborative links to Singapore, Chile, and Brazil.

Interestingly, England, Germany, Italy, the Netherlands, Japan, Australia, and China—all among the top 10 most productive countries—emerge as core members of the major collaborative clusters. This suggests that these nations not only lead in research output on ICU‐AW but have also established strong international research networks. The United States, while not prominently featured in the largest collaborative clusters, was an early contributor to the field and remains the most prolific publishing country. This early and sustained engagement likely reflects the nation’s advanced healthcare infrastructure, robust research funding, and high clinical awareness of ICU‐AW as a common and clinically significant complication among critically ill patients. In contrast, China initiated ICU‐AW research relatively late, with its first publication appearing in 2005—making it the latest entrant among the top 10 countries. Nevertheless, Chinese researchers have rapidly expanded their scholarly output, contributing 174 publications to date. This sharp upward trajectory underscores China’s growing commitment and intense research interest in ICU‐AW, highlighting its emergence as a key player in the global research landscape.

**Figure 3:**
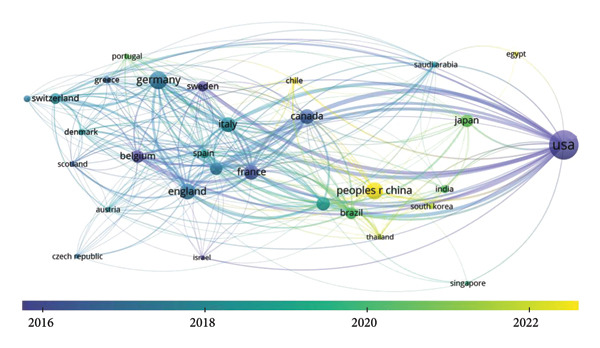
Cooperation network among all countries with time.

In VOSviewer, we applied a temporal overlay to the country collaboration network (Figure 3), where node colors reflect the average publication year of each country’s contributions: cooler tones (e.g., blue) indicate earlier research activity, while warmer tones (e.g., yellow) represent more recent engagement. The United States exhibits a predominantly blue‐colored node, confirming its early involvement in ICU‐AW research. In contrast, the nodes for China, Japan, and Brazil appear yellow, signifying their more recent entry into the field. Notably, China published its first ICU‐AW–related article in 2005, making it the latest starter among the top 10 most productive countries. Despite this delayed onset, China has demonstrated an exceptionally rapid increase in publication output, underscoring its dynamic and accelerating contribution to global ICU‐AW research.

### 3.3. Research Institution

Among the 1866 included publications, a total of 2657 research institutions contributed to the field of ICU‐AW. Table 2 lists the top 10 institutions by publication count, along with their betweenness centrality and country of affiliation. The University of Toronto ranked first with 43 publications, followed by Johns Hopkins University (41) and Karolinska Institutet (29). The University of Toronto also led in total citations and total link strength, indicating its mature research output and central role in the global ICU‐AW scholarly network—solidifying its position as a world‐leading institution in this field. Of the top 10 institutions, four are based in the United States and two in Germany, with the remaining four located in distinct countries. This distribution further underscores the United States’ pioneering role in ICU‐AW research, supported by a concentration of highly productive and well‐established academic centers. Notably, Karolinska Institutet (Sweden, 29 publications) and KU Leuven (Belgium, 22 publications) also feature among the top 10 despite their countries not ranking among the highest in overall national output. This highlights the exceptional focus, dedication, and research intensity of these individual institutions toward advancing knowledge on ICU‐AW.

**Table 2:** The top 10 institutions by documents.

Rank	Organization	Documents	Centrality	Country
1	Univ. Toronto	43	0.11	Canada
2	Johns Hopkins Univ.	41	0.11	United States
3	Karolinska Inst.	29	0.1	Sweden
4	Univ. Melbourne	29	0.08	Australia
5	Charité Univ. Med. Berlin	29	0.07	Germany
6	Univ. Kentucky	24	0.03	United States
7	Katholieke Univ. Leuven	22	0	Belgium
8	Humboldt Univ.	17	0.03	Germany
9	Univ. Washington	13	0	United States
10	Emory Univ.	12	0.03	United States

**Figure 4:**
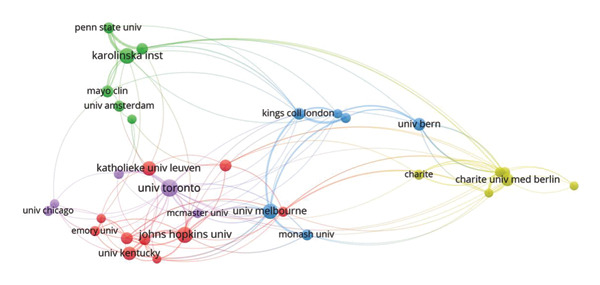
Cooperation network among all institutions.

To further explore institutional collaborations, we conducted a co‐occurrence analysis using VOSviewer, applying a minimum threshold of 15 publications per institution. This yielded a network of 33 institutions that met the criterion. These institutions were grouped into five distinct clusters, each represented by a different color in Figure 4. The largest cluster (red) comprises Emory University, Johns Hopkins University, KU Leuven, Massachusetts General Hospital, University of Brescia, University of Colorado, University of Kentucky, University of Washington, Vanderbilt University, and Wright State University. With the exception of KU Leuven (Belgium) and the University of Brescia (Italy), all other eight institutions are based in the United States, highlighting the dominant role of U.S. academic and medical centers in ICU‐AW research collaborations. The second‐largest cluster (green) includes Karolinska Institutet, Mayo Clinic, Penn State University, Sorbonne University, University of Amsterdam, and Uppsala University. Although the nodes in this cluster are relatively small—indicating modest individual publication outputs—the connecting lines between them are notably thick, reflecting strong and frequent collaborative ties among these institutions. This suggests a tightly knit research network characterized by high levels of cooperation despite lower overall publication volume.

**Figure 5:**
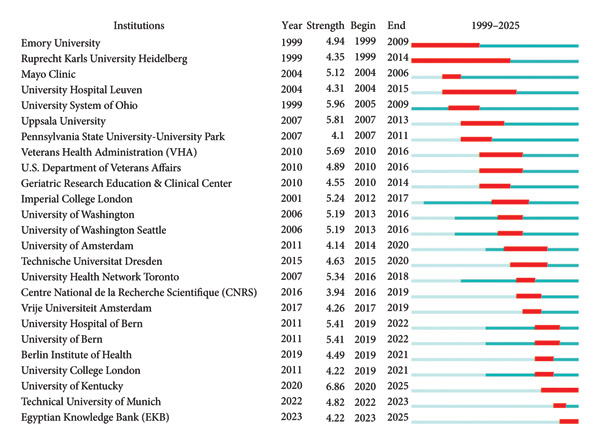
Top 25 institutions with the strongest citation bursts.

To further investigate the temporal dynamics and periods of intense research activity among these institutions, we performed a burst detection analysis using CiteSpace (Figure 5). The results reveal that Emory University (United States) and Heidelberg University (Ruprecht‐Karls‐Universität Heidelberg, Germany) were the earliest institutions to engage in ICU‐AW research. Moreover, they exhibited the longest‐lasting research bursts, indicating sustained scholarly activity and enduring influence in the field. In contrast, the University of Kentucky (United States), the Technical University of Munich (Germany), and the Egyptian Knowledge Bank (Egypt) represent more recent entrants into ICU‐AW research. Despite their later start, these institutions demonstrate strong citation bursts and notable publication output during their active periods, suggesting emerging prominence and significant potential for future contributions to the field.

### 3.4. Authors

The 1866 publications represent the collective contribution of 9016 authors. Table 3 lists the top 10 most prolific authors in terms of publication count. The top three authors are Lars Larsson (Sweden, 34 publications), Steffen Weber‐Carstens (Germany, 29 publications), and Greet Van den Berghe (Belgium, 28 publications). Among the top 10 authors, three are from the United States, each affiliated with different institutions. This indicates that numerous U.S. institutions have a strong interest in ICU‐AW research and host several leading researchers who are making significant contributions to the field.

**Table 3:** The top 10 authors by documents.

Author	Numbers	Country	Institution
Larsson, Lars	34	Sweden	Uppsala University
Weber‐Carstens, Steffen	29	Germany	Charité–Universitätsmedizin Berlin
Van den Berghe, Greet	28	Belgium	KU Leuven/University Hospitals Leuven
Needham, Dale M.	27	United States	Johns Hopkins University School of Medicine
Hermans, Greet	23	Belgium	KU Leuven
Morris, Peter E.	19	United States	University of Kentucky College of Medicine
Latronico, Nicola	18	Italy	University of Brescia
Wollersheim, Tobias	18	Germany	Charité–Universitätsmedizin Berlin
Horn, Janneke	17	Netherlands	Amsterdam University Medical Centers (University of Amsterdam)
Hough, Catherine L.	17	United States	University of Washington School of Medicine

**Figure 6:**
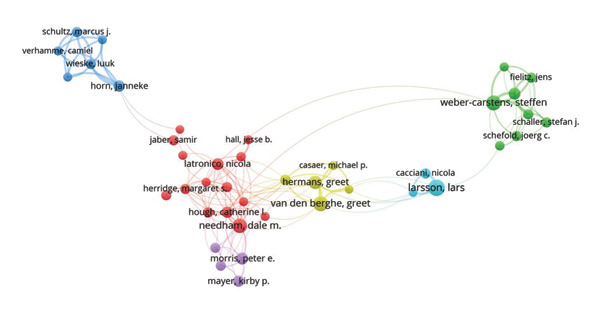
Cooperation network among all authors.

To further explore collaboration patterns among authors, we conducted a co‐authorship analysis using VOSviewer, setting a minimum threshold of 10 publications per author. Initially, 44 authors met this criterion; however, after excluding those with no co‐authorship links, the final network comprised 41 authors. These authors formed six distinct clusters, as illustrated in Figure 6. An examination of the number of clusters and the sparsity of inter‐cluster connections suggests that overall collaboration among leading authors in the field remains relatively limited. The largest cluster (red) consists of 15 authors, centered around Dale M. Needham and Catherine L. Hough—both based at different institutions in the United States. This reinforces the prominent role of U.S.‐based research teams in ICU‐AW scholarship and underscores the United States’ central position within the global collaborative network in this field.

**Figure 7:**
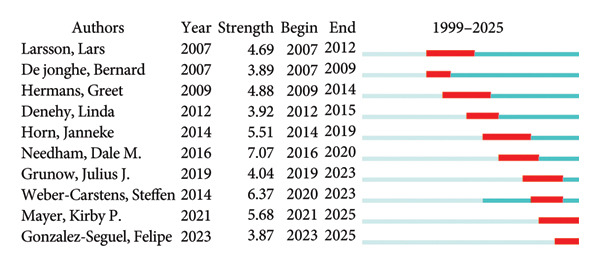
Top 10 authors with the strongest citation bursts.

Using CiteSpace, we performed a burst detection analysis to identify authors with periods of sharply increased publication or citation activity (Figure 7). The results highlight four researchers with notably high recent burst strength: Julius J. Grunow, Steffen Weber‐Carstens, Kirby P. Mayer, and Felipe Gonzalez‐Seguel. Investigating the publications of these authors may provide valuable insights into emerging research frontiers and evolving trends in ICU‐AW. Among them, Kirby P. Mayer is affiliated with an institution in the United States, and Felipe Gonzalez‐Seguel is based in Chile, while Julius J. Grunow and Steffen Weber‐Carstens are from Germany. The strong and sustained burst activity of German researchers further underscores Germany’s long‐standing commitment to ICU‐AW research and reflects a growing intensity of scholarly engagement in this field within the country.

### 3.5. Keywords

**Figure 8:**
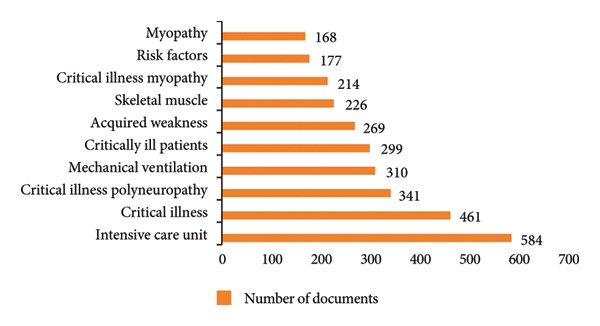
Top 10 keywords by frequency in ICU‐AW.

Figure 8 presents the top 10 keywords by frequency. Among them, terms such as “myopathy,” “critical illness myopathy,” “skeletal muscle,” “acquired weakness,” and “critical illness polyneuropathy” pertain to neuromuscular pathology and collectively reflect the core pathophysiological features of ICU‐AW. The keywords “critically ill patients,” “critical illness,” and “intensive care unit” highlight the clinical context and patient population most affected by this condition. Additionally, “risk factors” and “mechanical ventilation” appear prominently, indicating a growing research focus on modifiable contributors to ICU‐AW. This underscores an increasing awareness among scholars of the importance of identifying and mitigating high‐risk exposures—particularly prolonged mechanical ventilation—in the prevention and management of ICU‐AW.

**Figure 9:**
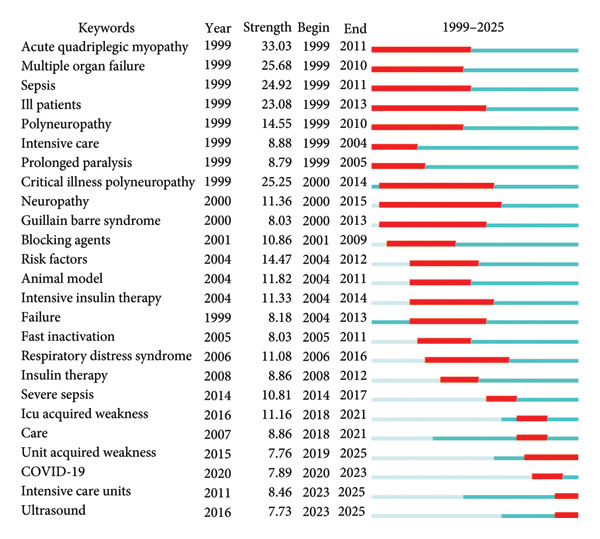
Top 25 keywords with the strongest citation bursts.

To examine the temporal dynamics and duration of research interest in key topics, we performed a keyword burst detection analysis using CiteSpace (Figure 9). Early and long‐sustained bursts were observed for terms such as “acute quadriplegic myopathy,” “multiple organ failure,” “sepsis,” “ill patients,” “polyneuropathy,” “intensive care,” “prolonged paralysis,” “critical illness,” “neuropathy,” and “Guillain–Barré syndrome.” These keywords reflect the foundational focus of early ICU‐AW research, which centered on underlying pathophysiology, systemic critical illness, and associated neuromuscular complications. Analyzing these enduring hot topics provides valuable insight into the historical trajectory and conceptual evolution of the field. In contrast, more recent bursts have emerged for terms including “unit acquired weakness,” “COVID‐19,” “intensive care units,” and “ultrasound.” Notably, the rising prominence of “ultrasound” after 2019 suggests a paradigm shift in research emphasis—from investigating etiology and risk factors toward early diagnosis, monitoring, and preventive strategies. This trend highlights the growing adoption of point‐of‐care ultrasound as a non‐invasive, real‐time diagnostic tool in the assessment of muscle structure and function among critically ill patients.

**Figure 10:**
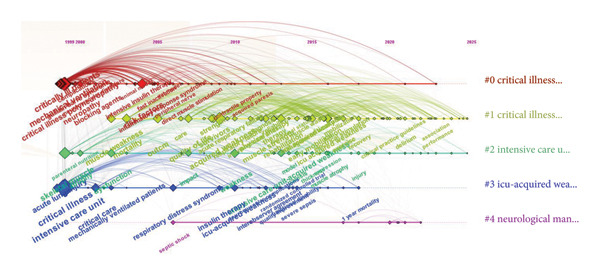
Timeline view of keywords related to ICU‐AW.

In CiteSpace, we conducted a timeline‐based keyword clustering analysis (Figure 10). The clustering parameters were set as follows: *k* = 8, node type = keyword, and time slice = 5 years. The results revealed five distinct clusters, with inter‐keyword connections visualized along a temporal axis. In this visualization, node size reflects keyword frequency, node color indicates publication time (with redder hues representing more recent years), and the horizontal position of each node corresponds to its first appearance. Cluster #0 (red) centers on “critical illness” itself, encompassing foundational concepts such as “critically ill patients” and “comorbidities,” thereby framing the overarching conceptual structure of the field. Cluster #1 (green) focuses on “outcomes,” linking terms including “mortality,” “quality of life,” and “acute respiratory failure,” highlighting research emphasis on prognostic evaluation in critically ill populations. Cluster #2 (blue) is anchored by “intensive care unit” and includes keywords such as “mechanically ventilated patients,” “respiratory distress syndrome,” and “septic shock,” reflecting a sustained interest in exploring the etiological and pathophysiological drivers of critical illness. Cluster #3 directly addresses ICU‐AW as a core entity, connecting keywords like “early mobilization,” “clinical practice guidelines,” “muscle atrophy,” and “rehabilitation,” thus tracing the evolution of the field from conceptual definition to evidence‐based interventions and guideline development. Cluster #4 (purple) emphasizes neuromuscular mechanisms and targeted interventions, featuring terms such as “neuromuscular electrical stimulation,” “peripheral nerve,” and “neuropathy,” which collectively probe the neurophysiological underpinnings of ICU‐AW and emerging therapeutic strategies.

## 4. Discussion

ICU‐AW is a multifactorial condition, with current evidence suggesting that both underlying patient comorbidities and clinical interventions during critical care are the primary contributors to its development [10]. Risk factors associated with pre‐existing conditions include hyperglycemia, sepsis, and multiple organ dysfunction, whereas those linked to clinical management predominantly involve mechanical ventilation, glucocorticoid administration, physical restraints, and other intensive care practices [11]. Several studies have further identified specific predictors of ICU‐AW, including advanced age, female sex, shock, an APACHE II score ≥ 15, and prolonged mechanical ventilation (≥ 48 h) [12, 13]. Additional significant risk factors encompass an ICU stay of ≥ 10 days, multiple organ dysfunction, acute kidney injury, severe sepsis [14, 15], hyperglycemia, elevated plasma osmolality, total parenteral nutrition for more than 3 days, sedation lasting over 2 days, external diaphragm pacing for more than 1 day, and the use of protective restraints exceeding 2 days (all *p* < 0.05) [16]. Notably, some investigators have proposed that glucocorticoid therapy administered for more than 3 days may reduce the incidence of ICU‐AW (*p* > 0.05) [17]; however, this finding remains contentious and warrants further validation.

The pathophysiological mechanisms of ICU‐AW remain only partially elucidated. Recent studies suggest that muscle atrophy and muscle dysfunction are the two principal contributors to ICU‐AW [18]. Muscle atrophy arises from neuroendocrine alterations that disrupt the balance between protein catabolism and anabolism, favoring net degradation [19]. In the ICU setting, prolonged bed rest—often necessitated by protective restraints and immobility—further exacerbates disuse atrophy of skeletal muscle fibers. Critically ill patients with respiratory failure frequently require extended mechanical ventilation, which itself may induce dysfunction of the diaphragm and intercostal muscles due to ventilator‐induced inactivity [20]. Moreover, prolonged controlled ventilation increases the risk of ventilator‐associated pneumonia (VAP), and both respiratory muscle atrophy and VAP have been implicated as pathogenic mechanisms in the development of ICU‐AW. Emerging evidence highlights a strong association between diaphragmatic dysfunction and ICU‐AW. Research teams have demonstrated that diaphragmatic impairment significantly elevates the risk of ICU‐AW, with a reported 2‐year survival rate of only 36% in affected patients, compared to 79% in those without ICU‐AW [21]. These findings underscore the pivotal role of diaphragmatic function not only in the pathogenesis of ICU‐AW but also as a key determinant of long‐term prognosis [22]. In recent years, interventions such as early mobilization and neuromuscular electrical stimulation (NMES) have shown promise in mitigating muscle deterioration and reducing ventilator dependence [23], thereby offering a potential strategy for the prevention of ICU‐AW [24, 25]. External diaphragm pacing—a form of NMES—induces repeated cycles of diaphragmatic contraction and relaxation, counteracting disuse atrophy [24]. Concurrently, fiberoptic bronchoalveolar lavage (BAL) effectively clears purulent secretions and inflammatory exudates that are difficult to expectorate, facilitating the removal of airway pathogens and inflammatory mediators. The combination of these two approaches may limit systemic absorption of inflammatory metabolites and toxins in the acute phase while accelerating the resolution of pulmonary inflammation. Notably, Chinese researchers have reported that the integrated application of external diaphragm pacing and fiberoptic BAL can significantly reduce alveolar pressure during positive‐pressure ventilation, improve diaphragmatic contractility, enhance respiratory muscle endurance, prevent diaphragmatic disuse, and promote recovery from lung inflammation—collectively reducing the incidence of ICU‐AW through improved pulmonary function. Importantly, this combined regimen has not been associated with an increased risk of complications, suggesting a favorable safety profile. Given its dual benefits in preserving diaphragmatic function and resolving airway inflammation, this multimodal strategy holds considerable promise for the prevention of ICU‐AW and warrants broader clinical implementation. Our bibliometric analysis reveals a growing research interest in ICU‐AW in recent years. The United States, Germany, China, England, and Italy have emerged as the most productive countries in this field. Notably, the United States exhibits the highest centrality, indicating its central and mature role in the global ICU‐AW research network. This leadership is further underscored by the fact that three of the top 10 most prolific authors originate from distinct U.S. institutions, reflecting sustained engagement by multiple research groups and institutions—likely a key driver of the country’s core status in this domain. These findings suggest that future collaborative studies on ICU‐AW could benefit from international partnerships with researchers in these leading countries to broaden the scope and generalizability of cross‐sectional investigations. China initiated its ICU‐AW research relatively late, with its first publication appearing only in 2005—making it the latest entrant among the top 10 most productive countries. Nevertheless, Chinese scholars have since published 174 relevant articles, demonstrating remarkable growth and strong research momentum. The volume of Chinese publications has surged in recent years, signaling an accelerating research trajectory. Given this rapid expansion, China is poised to make significant contributions to the field in the near future and stands out as a country with exceptional potential for advancing ICU‐AW research. Some Chinese research teams have found that traditional Chinese medicine treatment can improve muscle strength and daily living ability, shorten mechanical ventilation time, ICU length of stay, and total length of stay, reduce inflammatory factors, and improve the treatment effect of ICU‐AW. Traditional Chinese medicine treatment may be one of the future research directions for Chinese researchers [26]. Among research institutions, the University of Toronto stands out as a mature and globally leading center in ICU‐AW research. Emory University (United States) and Ruprecht‐Karls‐Universität Heidelberg (Germany) were among the earliest institutions to investigate ICU‐AW and have maintained sustained research activity over the longest period, reflecting enduring scholarly engagement in this field. More recently, the University of Kentucky (United States), the Technical University of Munich (Germany), and the Egyptian Knowledge Bank have emerged as active contributors. Despite their relatively recent entry, these institutions demonstrate notable strength in both publication output and citation burst intensity, suggesting high‐impact research trajectories. Future collaborative efforts may benefit from partnerships with these rising centers to foster innovation and broaden the global research network. Within the top 10 keywords identified in ICU‐AW literature [27], “risk factors” indicates that preventive research has reached a considerable level of maturity, while terms such as “critical illness polyneuropathy,” “skeletal muscle,” “critical illness myopathy,” and “myopathy” predominantly reflect a focus on pathophysiological mechanisms. Keyword co‐occurrence clustering further reveals a distinct blue cluster comprising “mechanically ventilated patients,” “respiratory distress syndrome,” and “septic shock,” which collectively emphasize etiological investigations of underlying critical conditions. Notably, emerging keywords—including “circulating lipids” and “diaphragmatic function”—are appearing with increasing frequency, potentially signaling novel mechanistic pathways in ICU‐AW pathogenesis. These concepts may offer promising avenues for advancing our understanding of disease mechanisms and informing targeted preventive and nursing interventions. Although research directly linking circulating lipids to ICU‐AW remains limited, extensive evidence supports their role in atherosclerosis—a condition that may indirectly contribute to the development of ICU‐AW [28].

As the top 10 keywords, rehabilitation training is of great significance for the treatment of ICU‐AW [29]. Rehabilitation training can improve the quality of life of critically ill patients admitted to the ICU at discharge. In clinical work, rehabilitation training should be paid more attention to. “Nursing” and “outcome” appeared early but recently showed a peak in citations [30]. From the appearance time and heat changes of these keywords, we can speculate that the research hotspots in the field of ICU‐AW research have shifted from the study of the disease itself to the outcome of the disease [31], but the study of the mechanism of the disease still needs researchers to continue to persist [32]. Before the new research breakthrough, clinical medical staff should do a good job in “nursing” and “rehabilitation.” Good nursing can prevent the occurrence of ICU‐AW, and correct rehabilitation treatment can improve the prognosis of ICU‐AW. Combined treatment, nursing, and rehabilitation can reduce the occurrence of ICU‐AW and promote pulmonary rehabilitation, thereby shortening the length of ICU stay [33]. To improve the quality of life of ICU patients in and out of hospital. A key limitation of this study is the temporal scope of the database, which may not capture the most recent contributions from authors, institutions, or evolving collaborative networks, thereby potentially underrepresenting the latest advancements in the field.

## 5. Conclusion

Through a bibliometric analysis of global research on ICU‐AW, this study delineates recent trends, leading countries and institutions, core research themes, and evolving directions in the field. These insights provide a robust evidence base to inform future international collaboration and academic exchange. In clinical practice, early identification and diagnosis of ICU‐AW—coupled with proactive mitigation of modifiable risk factors, implementation of multimodal therapeutic strategies, and intensified nursing and rehabilitation interventions—can promote recovery of diaphragmatic and pulmonary function. Such an integrated approach holds the potential to reduce the incidence of ICU‐AW, shorten ICU length of stay, and ultimately improve long‐term patient outcomes.

## Ethics Statement

This study is based on the analysis of publicly available data and does not involve human or animal experimentation; therefore, it qualifies for exemption from Institutional Review Board (IRB) approval.

## Consent

I confirm that I hold the copyright to this work and that I have the authority to grant permission for its publication. Any necessary permissions for the use of third‐party material have been obtained.

## Disclosure

All authors contributed to manuscript revision and read and approved the submitted version. The manuscript is original and has not been published elsewhere. It has not been submitted simultaneously for publication in any other journal or platform.

## Conflicts of Interest

The authors declare no conflicts of interest.

## Author Contributions

Wei Li and Jiadong Wang wrote the manuscript. Xi Feng revised the manuscript for intellectual content.

## Funding

This work was supported by Changsha Municipal Natural Science Foundation (kq2014013).

## Data Availability

The data that support the findings of this study are openly available in WOS at https://www.webofscience.com. The original contributions presented in the study are included in the article/Supporting Information. Further inquiries can be directed to the corresponding author.
